# P05.06 . “Empathy – a hands-on training”: format and evaluation of an experienced-based learning approach

**DOI:** 10.1186/1472-6882-12-S1-P366

**Published:** 2012-06-12

**Authors:** M Neumann, O Karnieli-Miller, H Goldblatt, D Tauschel, F Edelhäuser, G Lutz, C Scheffer

**Affiliations:** 1 University of Witten/Herdecke, School of Medicine, ICURAM, Herdecke, Germany; 2 University of Haifa, Department of Nursing, Haifa, Israel

## Purpose

Empathy-trainings, which are (a) theory-based, (b) for all healthcare professionals (HCPs), (c) for all stages of education/training and, (d) focused on experienced-based learning, are still rare. This German-Israeli study describes the format of an innovative empathy-training, incorporating these elements. Evaluations of eight trainings with integrative care students, nursing students, psychiatric rehabilitation practitioners and pharmacists are presented as well.

## Methods

A multidisciplinary group of integrative care teachers developed the training based on premises (a)-(d). After trainings, qualitative evaluations were conducted using an anonymous questionnaire containing five open-ended questions and questions on students’ personal data. Teachers wrote memos and some students wrote reflective essays about their training experiences.

## Results

The training consisted of six hands-on sessions: (1) What is clinical empathy? Participants’ empathic and non-empathic encounters in private life/ patients; (2) “Walking a mile in the patient’s shoes”: Finding one’s own path toward empathic understanding of patients; (3) "The art of empathy": Reflective art practices on empathy; (4) Non-verbal empathic communication: A photo safari; (5) Learning from patients about empathic communication; (6) Translating training experiences into practice. A theory handout on empathy was provided to participants, and discussed when needed. Each session included participants’ reflections on experiences.

Participants’ evaluations and essays and teachers’ memos revealed that students (N≈60) considerably appreciated the experienced-based learning approach, benefited from each exercise and stressed the importance of reflections. Teachers were impressed with students’ competence of developing their individual concept of empathy (Session 6). Evaluations suggest that this empathy-training is student-oriented, can easily be applied by teachers, used with all HCPs, in different cultural contexts, and and is suitable for inter-professional education.

## Conclusion

Training evaluations indicate that (1) experienced-based learning techniques can become catalysts to increase empathy and that (2) empathy is idiosyncratic and therefore can be best enhanced in finding one’s own empathic path, rather than only through abstract theory and communication-checklists.

